# Insights into the Complexities of Pharmacotherapy Parameters in Artificial Intelligence Models for Drug Selection, Precision Personalised Medicine and Optimal Therapeutic Outcomes

**DOI:** 10.3390/pharmaceutics18091157

**Published:** 2026-09-15

**Authors:** Malamati Kourti, Lefteris Zacharioudakis, Annita Kolnagou, Iliada Evripidou, George J. Kontoghiorghes

**Affiliations:** 1 Postgraduate Research Institute of Science, Technology, Environment and Medicine, Limassol 3021, Cyprus; m.kourti@euc.ac.cy (M.K.); ze00@aubmed.ac.cy (L.Z.); an.kolnagou@gmail.com (A.K.); dr_evripidou_iliada@hotmail.com (I.E.); 2 Department of Life Sciences, European University Cyprus, Nicosia 2404, Cyprus; 3 Department of Computer Science, American University of Beirut-Mediterraneo, Paphos 8046, Cyprus

**Keywords:** pharmacology, pharmacotherapy, pharmacogenomics, personalised medicine, artificial intelligence, input features and parameters, machine learning reliability

## Abstract

**Background**: Artificial intelligence (AI) is transforming pharmacology and pharmacotherapy by enabling the integration of large, heterogeneous datasets to support precision and personalised medicine. However, the reliability of AI-assisted therapeutic decision-making depends fundamentally on the selection, quality, and interpretation of pharmacological and clinical input features or parameters. Current AI models frequently overlook the multidimensional complexity of drug- and patient-specific factors, limiting their clinical applicability and generalisability. **Methods**: This narrative review was prepared based on 45 years of experimental work and relevant published research in drug design, development, and clinical experience in iron chelation therapy and personalised treatment approaches. Additional literature was identified through targeted searches of PubMed and Scopus using terms related to AI, machine learning, pharmacology, pharmacotherapy, and personalised medicine. Key pharmacological and clinical input features relevant to AI-assisted personalised drug selection include physicochemical drug properties, absorption, distribution, metabolism, excretion and toxicity characteristics, route of administration, drug interactions, pharmacokinetics, pharmacodynamics, multi-omics data, therapeutic efficacy, diagnostic biomarkers, statistical validation, governance, and explainable AI. A conceptual framework for AI-assisted personalised drug selection is also proposed as an example. **Results**: It is suggested that reliable AI-assisted pharmacotherapy requires the integration of diverse, interdependent datasets and parameters describing drug characteristics, patient variability, clinical outcomes, and real-world evidence. The incorporation of pharmacogenomics, electronic health records, diagnostic imaging and profiling, and validated computational descriptors can improve prediction of drug efficacy, toxicity, interactions, and therapeutic response. Furthermore, robust model validation, data governance, cybersecurity, and continuous monitoring are identified as essential prerequisites for safe clinical implementation. The proposed conceptual framework illustrates how clinical admissibility filtering, model-based ranking, local attribution, and clinically supervised decision support may enhance personalised drug selection while maintaining human oversight. **Conclusions**: Artificial intelligence has considerable potential to improve drug selection and personalised pharmacotherapy. However, its success depends on comprehensive integration of pharmacological knowledge with high-quality clinical data, rigorous validation, and responsible governance. The multidimensional framework presented here provides a foundation for the potential development of clinically interpretable, reliable, and patient-centred AI systems capable of supporting safer and more effective precision medicine.

## 1. Introduction

The introduction and development of artificial intelligence (AI) tools are currently affecting many different sectors of everyday life, including rapid access to widely available published information and its use in supporting research in many fields of pharmacology, pharmacotherapy and medicine [1,2,3,4]. However, in many cases, and particularly in medicine and pharmacotherapy, the practical use of AI tools remains cautious because of concerns regarding reliability, transparency, explainability, clinical accountability, and trust in the responsible use of AI in healthcare [5,6]. Different AI technologies also present separate limitations and risk profiles. For example, predictive machine learning (ML) models may be affected by issues such as bias, overfitting, data quality, and limited generalisability, whereas generative AI systems, including large language models (LLMs), may produce inaccurate or fabricated information. Furthermore, the use of AI-derived information may be associated with risks when involved in critical, life-saving or life-threatening decisions, which may have major consequences for public health, clinical practice, and the planning of clinical and pharmacological research. In this context, the selection, evaluation and secure handling, integrity, and reliability of pharmacological and clinical data used by AI systems become critical, as insufficient, compromised, or manipulated data may directly affect therapeutic decisions and patient safety.

The rapid pace of AI development is currently unpredictable and may, among other effects, lead to inaccuracies, which are critical for patients’ health and medical care in general. At this stage, AI should generally be considered as a supportive or advisory tool in pharmacology, pharmacotherapy, and medicine, as well as in other related sciences, and its outputs should be evaluated and verified using professional knowledge and experience. This requirement also extends to ensuring the security, traceability, and auditability of AI-generated recommendations, particularly when such systems are used in clinical or pharmacotherapeutic decision support. Emerging agentic AI systems may introduce additional challenges related to autonomous task execution and system oversight. In particular, the input of clinical and pharmacological features and parameters is critical for personalised medicine. In this context, the diversity and complexity of input features and parameters required for application in personalised medicine models across all diseases have not yet been determined. This underlines the limitations of AI predictive analytics in capturing the full range of each patient’s pharmacological and clinical characteristics, which are essential for minimising clinical errors and optimising therapeutic outcomes.

The main aim of this work is to introduce basic but important input features and parameters in the fields of pharmacology and pharmacotherapy, which are required to highlight the need for a broader and deeper understanding of drug selection and use within the framework of optimal precision in personalised therapy. Particular emphasis is placed on pharmaceutics-related determinants of therapeutic outcomes, including drug physicochemical properties, formulation parameters, routes of administration, ADMET (absorption, distribution, metabolism, excretion, toxicity) characteristics, pharmacokinetic and pharmacodynamic variability, and other factors influencing personalised drug selection and optimisation. Another aim is to present new strategies and to highlight emerging issues related to drug parameters to be used in ML in order to gain a wider and deeper insight into drug design and drug application. This includes new concepts and factors influencing drug action, risk–benefit assessment, dosing regimens, drug interactions, and many other pharmacological features, all of which could influence optimal drug use and therapies in the context of personalised medicine [7,8,9,10]. Special attention will also be given to the secure use of sensitive pharmacological and patient-related data within ML workflows, ensuring data confidentiality, integrity, and controlled access.

A secondary aim of this work is to identify limitations, misconceptions, external influences, drawbacks, and other effects related to the use of AI, including risks associated with data quality, model manipulation, and security vulnerabilities, which may also affect drug activity and overall therapeutic outcomes in personalised medicine. Such approaches may also contribute to improved AI-assisted drug design and targeting, as well as to safer and more effective drug use, ultimately enhancing therapeutic interventions and increasing precision within personalised medicine [11,12,13,14].

In this study, a conceptual framework is proposed that brings together the pharmacological, biological and patient-specific factors and features influencing drug response into a single structured model. The primary focus of this review is to gather important features and parameters for the construction of AI-assisted personalised drug selection and therapeutic decision support models. Treatment-response prediction, risk-benefit assessment, and dose optimisation are also considered important complementary components that contribute to the broader goal of selecting the most appropriate therapeutic option for an individual patient. The proposed framework is intended for clinical scenarios in which therapeutic outcomes may be influenced by multiple interacting pharmacological, clinical, genomic, or biomarker-related factors, particularly where individual variability may affect efficacy, toxicity, or treatment response. By integrating these interconnected parameters, the framework aims to provide a basis for developing more transparent, reliable, clinically relevant and explainable AI systems to support personalised drug selection.

The instigation of this review was driven by the need to develop personalised medicine models aimed at improving therapeutic outcomes, following more than 45 years of research experience in drug design, development, and clinical application, particularly in the field of iron and other metal chelation therapy [15,16,17,18,19,20]. Within this context, a large number of physicochemical, pharmacological, clinical, and other variables, together with interactions involving drugs, nutrients, and endogenous biomolecules, were found to play major roles in determining the efficacy and toxicity of chelating and other therapeutic agents and drugs [21,22,23,24,25,26]. Furthermore, advances in diagnostic technologies, such as the magnetic resonance imaging (MRI) technique T2*, have made it possible to monitor and measure excess iron levels in different organs, thereby enabling the assessment of treatment efficacy and the optimisation of chelating drug dosing in individual patients through personalised therapeutic approaches [27,28,29,30]. Overall, the selection of the literature included in this narrative review was primarily guided by the authors’ expertise and practical experience in pharmacology, pharmacotherapy, and personalised medicine [8,10,19,31,32,33,34,35]. However, we acknowledge that this selection strategy may under-represent topics outside the authors’ primary areas of expertise.

To complement this expert-driven approach to chelating and other drugs and to propose a possible concept of constructing relevant AI models, targeted searches in PubMed and Scopus were also conducted between January and May 2026 using combinations of terms related to AI, ML, pharmacology, pharmacotherapy, personalised medicine, pharmacogenomics, drug discovery, drug selection, pharmacokinetics, pharmacodynamics, ADMET, explainable AI, clinical decision support systems, and precision medicine. Articles were selected according to their relevance to the scope of the review, with emphasis on pharmacological determinants of drug response, AI-assisted decision support, pharmacogenomics, clinical applications of ML, and related methodological considerations. Both primary research and review articles were included to provide a balanced overview of foundational concepts, emerging developments, current challenges, and clinically relevant perspectives.

## 2. The Complexity of Input Features in the Design of AI-Assisted Models in Personalised Medicine

There is vast complexity and variability in the input features and parameters needed for the design of an AI-assisted model in personalised medicine for each disease, each patient, and each drug. In each disease, drug case, and patient, different characteristics and conditions apply, which have to be selected and ranked according to their impact and priority, potentially leading to an AI-assisted model of optimised drug treatment for each individual in personalised medicine [8,10]. In this context, an AI-assisted personalised drug selection programme could be investigated in each disease based on the most important and widely used measurable physicochemical and pharmacological parameters, as well as the patient’s demographic, genetic, physical, underlying condition and other characteristics.

Considering that AI is based on mathematical models involving algorithms, the selection and quality of the input variables are important for AI-assisted personalised drug selection programmes. In this context, the inclusion of clinically relevant and informative variables may improve the ability of a model to characterise individual patients and predict therapeutic outcomes. However, increasing the number of variables alone does not necessarily improve model performance and may introduce additional complexity, requiring appropriate feature selection, validation, and model optimisation. Similarly, the larger the number of patients undertaking the same treatment under comparable conditions, the greater the potential reliability and generalisability of efficacy and safety assessments.

**Figure 1:**
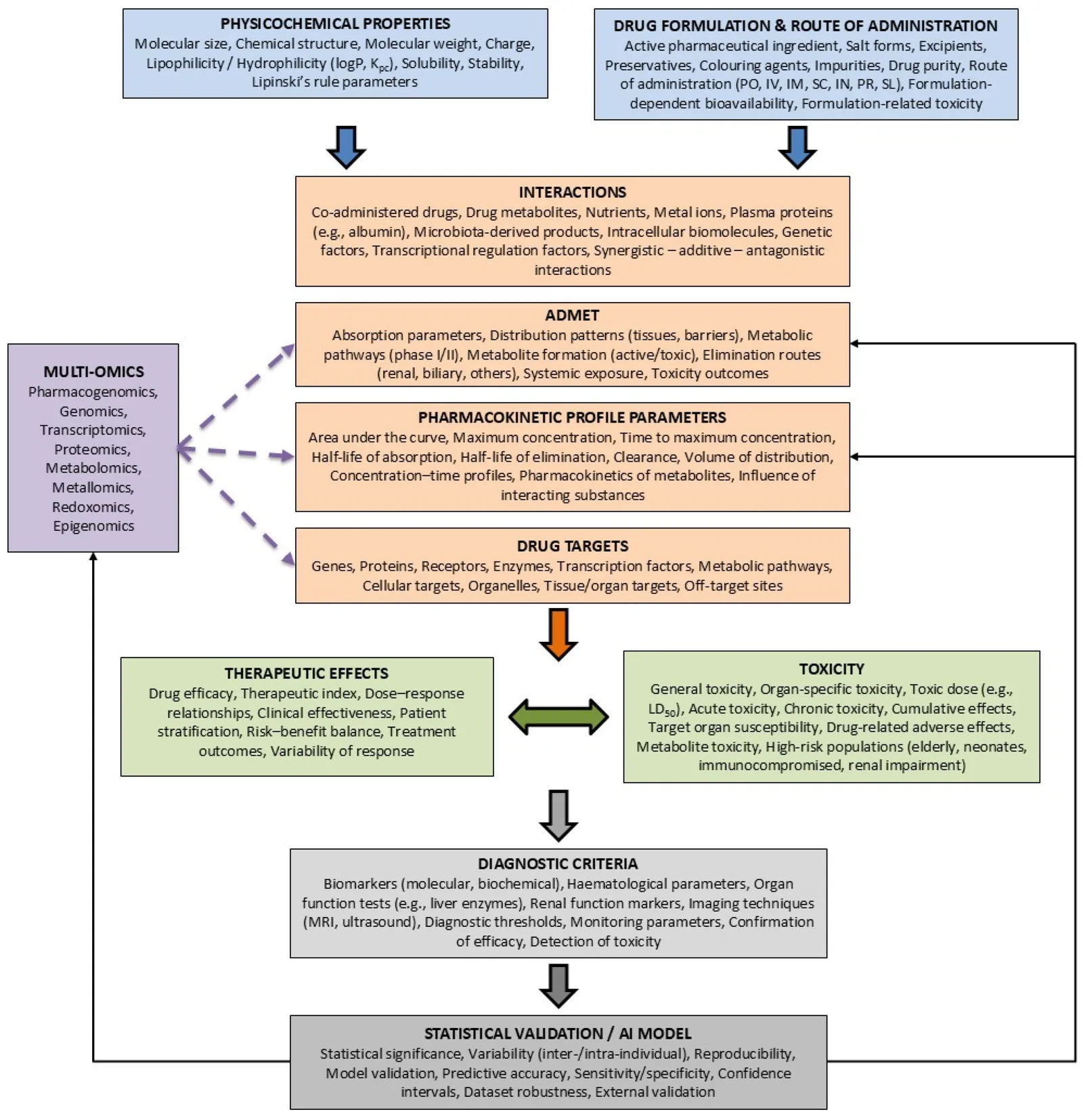
Integrated framework for AI-driven personalised drug selection. A multi-layered framework integrating drug-specific properties, biological processes, clinical outcomes, and model validation is depicted as affecting personalised medicine. Physicochemical properties and drug formulation/route of administration determine initial drug behaviour and feed into drug interactions. These, together with patient-specific factors, shape the central ADMET and pharmacokinetic processes, as well as drug target engagement. Multi-omics data modulate these mechanisms by capturing inter-individual variability. Downstream, therapeutic effects and toxicity represent clinical outcomes and jointly define the risk–benefit balance. Diagnostic criteria provide measurable confirmation of efficacy and toxicity and feed into statistical validation and AI modelling for prediction and optimisation. (Arrows: Solid downward arrows indicate the primary causal flow from drug properties to clinical outcomes. Bidirectional arrows between therapeutic effects and toxicity represent their dynamic interplay. Dashed arrows from the multi-omics layer indicate modulatory effects on ADMET, pharmacokinetics, and targets. Feedback arrows from the statistical validation/AI layer denote iterative model refinement based on clinical data). (Abbreviations: ADMET: absorption, distribution, metabolism, excretion, toxicity, IM: intramuscular, IN: intranasal, IV: intravenous, K_pc_: lipid/water partition coefficient, LD_50_: lethal dose causing 50% mortality, logP: logarithm of the partition coefficient, MRI: magnetic resonance imaging, PO: oral, PR: per rectum, SC: subcutaneous, SL: sublingual).

Many variants and input features are generally involved in AI-assisted personalised drug selection. In particular, a comprehensive framework for AI-assisted personalised drug prescribing must integrate multiple layers of pharmacological information, organised from drug-specific properties to clinical outcomes and model validation, as illustrated in the list of parameters, features and variants provided in Figure 1. It should be noted that each group of features or parameters contributes distinct but interconnected information that collectively determines the level of therapeutic efficacy and safety of an administered drug for each individual patient.

## 3. Physicochemical Properties, Drug Formulation and Route of Administration Factors and Parameters Affecting Pharmacological Activity

In general, the structural features and molecular aspects of drugs largely determine their physicochemical, pharmacological, and toxicological properties. Similarly, the formulation and route of administration of drugs influence their efficacy and toxicity. These and many other parameters could be utilised in ML processes for AI in designing predictive models for personalised medicine.

At the input level in particular, physicochemical properties such as molecular size, structure, charge, lipophilicity/hydrophilicity, solubility, and stability play a fundamental role in drug behaviour (Figure 1). These properties influence membrane permeability, plasma protein binding, tissue distribution, and the ability of the drug to interact with biological targets [36]. Established criteria, such as Lipinski’s rule of five, provide a framework for assessing drug-likeness and identifying compounds that may present poor absorption or permeability following oral administration [37,38]. In parallel, drug formulation and route of administration further modulate drug exposure. Pharmaceutical factors, including salt forms, excipients, preservatives, impurities, and overall drug purity, can significantly alter drug dissolution, absorption, efficacy, and toxicity profiles [39,40,41,42]. The route of administration (e.g., oral (PO), intravenous (IV), subcutaneous (SC), intramuscular (IM), intranasal (IN), per rectum (PR) (suppository), and sublingual (SL) determines the initial pharmacokinetic path and can lead to substantial differences in bioavailability, organ targeting, and systemic exposure [43].

A clinically relevant example of the importance of drug formulation is paclitaxel, where formulation strongly influences both tolerability and therapeutic performance. Conventional solvent-based paclitaxel requires solubilizing agents, whereas nanoparticle albumin-bound paclitaxel was developed as a solvent-free formulation. In a phase III trial in metastatic breast cancer [44], albumin-bound paclitaxel showed higher response rates and longer time to tumour progression than standard solvent-based paclitaxel, with no hypersensitivity reactions reported despite the absence of routine premedication. This example shows how formulation can modify drug delivery, the safety profile, and clinical response. Another example is amphotericin B, where a lipid-based drug formulation has substantially changed clinical use. Liposomal amphotericin B has shown similar antifungal efficacy to conventional amphotericin B, but with significantly fewer infusion-related reactions and lower nephrotoxicity [45]. This demonstrates how reformulation of the same active drug can improve the risk–benefit profile, particularly in vulnerable patients.

Another example is the commonly used drug omeprazole, which is an enzymatic proton pump inhibitor, unstable under acidic gastric conditions and therefore requires enteric-coated formulations or stomach acid neutralizers as alternatives to prevent degradation prior to absorption. These formulation strategies ensure drug release in the more neutral pH of the small intestine, thereby enabling higher absorption, effective systemic exposure and therapeutic efficacy [46,47,48]. A further similar example is enalapril, which is an angiotensin-converting enzyme inhibitor used for lowering blood pressure. Enalapril was developed as a prodrug to overcome the poor oral bioavailability of the active compound enalaprilat. By increasing lipophilicity through esterification, enalapril achieves improved gastrointestinal absorption and is subsequently converted to the active form in vivo, demonstrating how physicochemical optimisation can enable effective clinical use [49].

Route of administration can also directly affect prescribing decisions. In human epidermal growth factor receptor 2 (HER2)-positive breast cancer, subcutaneous trastuzumab demonstrated non-inferior pharmacokinetics and pathological complete response compared with intravenous trastuzumab in a phase III trial, while offering a simpler mode of administration [50]. Such examples show why physicochemical properties, formulation, and administration route should be considered key input variables in AI-driven models for personalised drug prescribing.

Organ targeting, efficacy, posology, time course of treatment, compliance, tolerance, cost, and emergency treatments are some other features and parameters influencing drug selection, route of administration, and personalised medicine requirements [8,10,51,52,53,54,55]. For example, in iron chelation therapy for the treatment of transfusional iron overload, the daily administration of the chelating drugs deferoxamine (DF), deferiprone (L1), and deferasirox (DFRA) is required to eliminate the excess iron accumulated in various organs from chronic red blood cell transfusions. Deferoxamine is orally inactive and administered subcutaneously (SC) over several hours using a pump because of its rapid blood clearance. In contrast, the lipophilic orally active drug DFRA is administered once daily because of its slow clearance and possibility of drug accumulation toxicity, whereas the hydrophilic orally active drug L1 is usually administered 2–3 times daily [51,52,53,54,55]. Most patients cannot tolerate the DF injections and use the oral drugs. However, in cases of toxicity or intolerance to any of the three drugs, the patients can switch to the other drugs. Chelating drug combinations are usually more effective in eliminating excess iron than monotherapy. In particular, L1 and its combination with DF have been shown to be the most effective in eliminating excess iron from the heart, which is the target organ responsible for the increased mortality observed in transfusional iron-overloaded patients [56,57,58,59,60]. Similarly, personalised protocols for this combination have been shown to eliminate all excess iron from the body, achieving complete therapy for iron overload in transfusional iron-overloaded patients [61,62,63]. Lower doses of L1, in particular, are sufficient for maintaining normal iron levels in most transfused patients who have achieved normal iron stores in the liver, spleen, and heart [51,63,64].

The safety, efficacy, and ability to cross the blood–brain barrier in the case of L1 are currently the subject of repurposing of the drug in many diseases, including neurodegenerative diseases and cancer [60,65,66]. Similarly, many other administration routes have also been tested for DF, where IN administration of DF may be examined for different brain disease applications [67,68,69,70,71,72].

## 4. Drug Interaction Effects and Related Pharmacological Features

**Figure 2:**
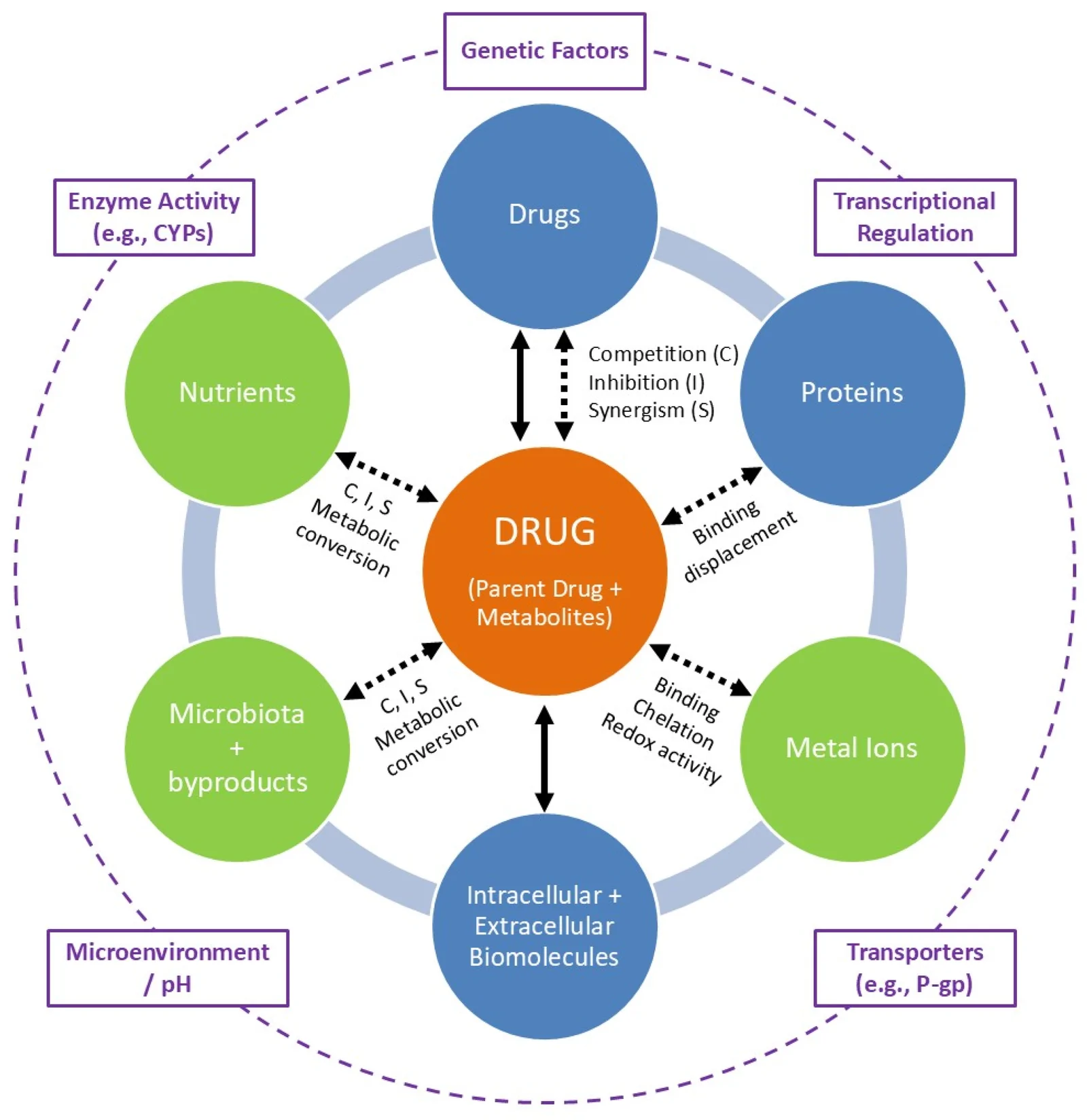
Multidimensional network of drug interactions and modulatory influences. A radial interaction framework is depicted, illustrating how a drug and its metabolites interact with multiple biological and external components. Direct interaction nodes include co-administered drugs, proteins, and extracellular and intracellular biomolecules, while system-level factors such as nutrients, microbiota, and metal ions further influence drug behaviour. These interactions occur through mechanisms including inhibition, competition, synergism, binding, displacement, metal chelation, and metabolic conversion. Outer modulatory factors, including genetic variation, transcriptional regulation, enzyme activity, transporters, and microenvironment characteristics, could modify these interactions and contribute to inter-individual variability. (Arrows: Solid arrows indicate direct interactions with the drug. Bidirectional arrows denote reciprocal effects between interacting components. Dashed arrows represent modulatory influences exerted by systemic and patient-specific factors. Interaction labels on arrows specify the underlying mechanism in each case). (Abbreviations: C: competition, CYP: cytochrome P450, I: inhibition, P-gp: P-glycoprotein, S: synergism).

A large number of upstream features, including many factors and properties, directly influence the drug interaction layer, where the drug and its metabolites interact with co-administered drugs, nutrients such as ascorbic acid and other vitamins, metal ions, plasma proteins such as albumin, microbiota-derived metabolites and by-products, and extracellular and intracellular biomolecules, all of which influence the efficacy and toxicity of a drug in the context of personalised medicine [7,72,73,74,75,76,77]. Such interactions may alter drug absorption, metabolism, or target binding through mechanisms such as enzyme inhibition, transporter competition, or receptor-level modulation [78,79]. Genetic and transcriptional factors further modify these processes, contributing to inter-individual variability in drug response [80,81,82] (Figure 2).

There are many examples of interactions of drugs and their metabolites at various levels, including those described in Figure 2. Ascorbic acid, or vitamin C, has been shown, for example, to increase iron absorption in iron deficiency, whereas in combination with DF it increases iron excretion in iron-loaded patients [35,83,84,85,86,87]. Another clinically relevant example is the interaction between grapefruit juice and simvastatin. Grapefruit juice inhibits intestinal CYP3A4-mediated first-pass metabolism and can markedly increase systemic exposure to simvastatin and its active metabolite, thereby increasing the potential for both therapeutic and adverse effects. This example shows how dietary components can substantially alter drug bioavailability and should therefore be considered in personalised prescribing models [88,89].

Drug–drug interactions are equally important in clinical decision-making [7]. For example, co-administration of amiodarone with warfarin increases the anticoagulant effect of warfarin and often requires closer international normalised ratio (INR) monitoring and dose adjustments. This interaction is particularly relevant because warfarin has a narrow therapeutic index, meaning that relatively small changes in exposure can increase bleeding risk or reduce anticoagulant efficacy [90,91]. Similarly, clopidogrel response can be affected by both simultaneous medications and genetics, as CYP2C19 loss-of-function variants and CYP2C19-inhibiting proton pump inhibitors, such as omeprazole, may reduce formation of the active clopidogrel metabolite and alter cardiovascular outcomes [92,93,94].

Interactions with metal ions also provide a clear example of how chemical interactions at the molecular level can affect treatment. Tetracyclines, anthracyclines, catecholamines, hydroxyurea, aspirin, and many other classes of drugs or their metabolites are known to interact with iron and other metal ions, affecting their efficacy and toxicity [75,95,96,97,98]. Ciprofloxacin, for example, forms complexes with iron and other multivalent cations, reducing its gastrointestinal absorption and lowering systemic exposure. This type of interaction is clinically important because it may reduce antibiotic efficacy if administration times are not appropriately separated [99,100].

Finally, the gut microbiota and its by-products represent an additional interaction layer that is increasingly relevant to personalised pharmacology [101,102,103,104,105,106,107,108,109]. For example, the cardiac drug digoxin can be inactivated by the gut bacterium *Eggerthella lenta*, and microbial genes involved in this process may predict the extent of drug inactivation. Dietary factors can further modify this interaction, showing how the microbiota and its by-products, nutrients, and drug metabolism can converge to influence patient-specific drug exposure pharmacology [101,102,103,104,105,106,107,108,109,110,111]. Together, these examples show that drug interaction data should be incorporated into AI-based ML models, particularly when interactions affect drugs with narrow therapeutic indices, variable metabolism, or strong dependence on the patient-specific biological context.

## 5. Consideration of Factors Related to Drug Absorption, Distribution, Metabolism, Excretion, and Toxicity

The therapeutic activity of each drug is largely dependent on its pharmacological properties and other features. At the core of the pharmacological framework lies the ADMET axis, which integrates the absorption, distribution, metabolism, elimination, and toxicity properties of a drug (Figure 1). This axis determines the overall fate of both the parent drug compound and its metabolites, including the formation of active or toxic intermediates through phase I and phase II metabolic reactions. Phase I metabolism generally involves oxidation, reduction and hydrolysis reactions, whereas phase II metabolism consists of conjugation reactions that increase the water solubility of drugs and their metabolites, thereby facilitating excretion. Since both phases exhibit considerable inter-individual variability, they represent important pharmacological predictors that should be considered in AI-based models for predicting therapeutic efficacy, toxicity and optimal dosing in personalised medicine [79]. Closely linked to ADMET are pharmacokinetic parameters, which provide quantitative descriptors of drug exposure, including AUC (area under the curve), C_max_ (maximum concentration), T_max_ (time to maximum concentration), half-lives of absorption and elimination, clearance, and volume of distribution. These parameters capture time-dependent concentration profiles and are essential for predicting dose–exposure–response relationships [79,112].

In addition to iron or other metal ion interactions with drugs described above [75,95,96,97,98], a clinically relevant example of variability in drug absorption is levothyroxine, where gastrointestinal conditions and co-administration with food, calcium supplements, or proton pump inhibitors can significantly alter bioavailability [113,114]. This variability often necessitates careful timing of administration and dose adjustment to achieve stable thyroid hormone levels.

Drug distribution can also significantly affect therapeutic outcomes, particularly for highly protein-bound drugs. For example, phenytoin is extensively bound to plasma proteins, and only the unbound fraction is pharmacologically active. In conditions such as hypoalbuminemia or renal impairment, the free fraction of phenytoin increases, potentially leading to toxicity despite normal total plasma concentrations. This supports the need for free drug monitoring in selected patients in the context of personalised medicine [115].

Drug metabolism represents another major source of inter-individual variability, often driven by genetic factors. A well-established example is codeine, which requires metabolic activation by the enzyme CYP2D6 to form morphine. Patients with reduced CYP2D6 activity may experience insufficient analgesia, while ultra-rapid metabolizers may be at increased risk of opioid toxicity [116,117,118]. In another example, an exception in glucuronidation was observed in one patient treated with the chelating drug L1, whereas in hundreds of other patients in similar studies, monitoring of L1 glucuronidation proceeded normally [119]. These examples highlight the need for and importance of considering metabolic pathways in personalised prescribing and monitoring metabolic changes at a personal level.

Elimination routes and processes, particularly renal clearance, are also critical determinants of drug exposure and safety. For instance, methotrexate is primarily eliminated through the kidneys, and impaired renal function can lead to drug accumulation and severe toxicity. As a result, careful dose adjustment and monitoring are required in patients with reduced renal function [120,121]. A similar elimination process of almost exclusive renal clearance applies to the chelating drug L1 [119,122,123]. In this case, an increase in plasma aluminium concentration was observed in renal dialysis patients treated with L1, which was subsequently eliminated as an L1-aluminium complex during haemodialysis [124,125].

Together, the above and many other examples show how variability across ADMET processes, including interactions with metal ions, nutrients, proteins, and microbiota, can affect drug exposure, efficacy, and safety. These processes are often interdependent, as, for example, changes in metabolism or elimination may alter toxicity profiles. Hence, the need to integrate pharmacokinetic and patient-specific data into AI-driven models for personalised pharmacotherapy is further reinforced.

## 6. Parameters and Factors Related to Drug Targets and Therapeutic Response

Beyond pharmacokinetic processes, therapeutic response is ultimately determined by drug–target interactions. Drug targets represent the biological sites of action, including genes, proteins, receptors, enzymes, and entire metabolic pathways at the cellular or organ level. Effective therapy depends on selective and adequate target engagement, while off-target interactions may contribute to adverse effects [126,127,128]. Importantly, this mechanistic core is modulated by multi-omics data, including pharmacogenomics, transcriptomics, proteomics, metabolomics, metallomics, redoxomics, and epigenomics [125,129,130,131,132]. These layers capture patient-specific biological variability and can explain differences in drug metabolism, target sensitivity, and susceptibility to toxicity [128,133,134,135,136,137].

The increasing availability of large-scale genomic resources, building on the Human Genome Project and subsequent population-based initiatives, has enabled the systematic identification of genetic variants such as single nucleotide polymorphisms (SNPs) associated with disease susceptibility and drug response [138]. These variants can affect the presence and function of drug targets, thereby directly informing and facilitating therapeutic selection [138]. Large-scale population genomics initiatives further highlight the extent of inter-individual genetic variability. For example, the All of Us Research Program [139] has generated extensive genomic and health-related data from diverse populations, demonstrating significant variability in genetic variants associated with disease susceptibility and drug response. Such datasets provide an important resource for identifying clinically relevant targets and biomarkers. Therefore, they can be integrated into AI-driven models to enhance patient stratification and optimise personalised therapeutic selection.

A clear example of the importance of target availability in therapeutic decision-making is provided by breast cancer subtypes. Hormone receptor-positive and HER2-positive breast cancers express defined molecular targets, such as the oestrogen receptor (ER) or HER2. This enables the use of targeted therapies, including endocrine treatments, such as tamoxifen or aromatase inhibitors, and anti-HER2 agents, such as trastuzumab or pertuzumab [140,141,142,143,144]. In contrast, triple-negative breast cancer (TNBC), defined by the absence of ER, progesterone receptor, and HER2 overexpression, lacks these established therapeutic targets and is therefore not responsive to standard hormonal or HER2-directed therapies. As a result, treatment has historically relied primarily on cytotoxic chemotherapy, illustrating how the absence of specific targets limits therapeutic precision and contributes to poorer clinical outcomes [145,146]. This example highlights the critical role of genomic and molecular profiling in identifying actionable targets and also underscores the need to integrate high-dimensional biological data into personalised treatment strategies.

Another major example of variability in therapeutic targeting involves the haemoglobinopathies, which are the most common group of genetic disorders in humans. It is estimated that there are more than 1800 human haemoglobin variants [147]. Most of these variants arise from single amino acid substitutions in the α and β globin chains, which, in many cases, cause no functional difficulties in oxygen transport by haemoglobin, whereas in a few cases, such as in sickle cell anaemia, the single amino acid substitution abnormality in haemoglobin causes serious side effects and requires treatment for life [148,149,150,151,152]. Similarly, abnormalities in the rate of production of α and β globin chains lead to the other major group of haemoglobinopathies, namely thalassemia. For example, in thalassemia, major haemoglobin is not functional, and lifelong red blood cell transfusions from normal blood donors are, in most cases, required for affected patients to survive [149,150,153]. Different therapeutic options are available based on a risk/benefit assessment and quality of life parameters and factors in each patient case, both in thalassemia major and sickle cell disease. In the majority of patients in both haemoglobinopathy categories, long-term survival was achieved by treating the symptoms. For example, in the vast majority of thalassemia major patients, effective iron chelation protocols are used for the elimination of iron overload caused by chronic transfusions, whereas complete therapy may be achieved by bone marrow transplantation from a compatible sibling, which is mostly available for younger patients. Gene therapy is also available but is still too risky and very expensive for the vast majority of thalassemia patients [62,63,64,73,154,155,156].

The variability of therapeutic options and the risk/benefit assessment for their use in selected patients with haemoglobinopathies and other diseases highlight the need for an individualised therapeutic approach. Such approaches, including the characterisation of clinical and pharmacological variables, could potentially lead to the design of AI-driven models for personalised medicine and optimised therapy for each patient.

## 7. Therapeutic Effects and Toxicity Factors in Personalised Medicine

Therapeutic drugs are, in most cases, approved by the drug regulatory authorities for the treatment of diseases based mainly on the findings of appropriate clinical trials. In such cases, treatment with the candidate drug could show, for the majority of patients, that the benefits are higher than the risks in comparison to other drugs or a placebo [55,157]. Usually, in clinical studies involving new drugs, the causes of variations, such as lower efficacy and higher toxicity observed in some of the treated patients, are not investigated. In this context, there are many factors and parameters that could influence the efficacy and toxicity of drugs in individual patients [43,158,159,160,161,162,163,164]. Ideally, both the general and individual variations in the therapeutic characteristics affecting each treated patient with a specific drug have to be investigated and specified in the context of personalised medicine.

In therapeutic drug interventions, the clinical outcomes are defined by the balance between therapeutic effects and toxicity (Figure 1). Therapeutic variables include drug efficacy, dose–response relationships, therapeutic index, and variability in clinical response across patient populations. In contrast, toxicity encompasses general and organ-specific adverse effects, dose-dependent toxicity thresholds, and both acute and chronic outcomes. Particular attention is required for high-risk populations, such as elderly individuals, neonates, pregnant women, immunocompromised patients, and those with renal or hepatic impairment, where altered physiology may significantly affect drug handling and safety [165,166,167].

A well-established example of the balance between efficacy and toxicity is warfarin, which has a narrow therapeutic index and requires careful dose titration to maintain therapeutic anticoagulation while avoiding bleeding complications. Small variations in dose, diet, co-medication, or genetic factors can significantly alter anticoagulant response, necessitating regular monitoring of the INR to optimise the risk/benefit balance [80,90]. Warfarin also represents one of the most well-studied examples of personalised pharmacotherapy. Clinical and pharmacogenomic factors, including variants in *VKORC1* (vitamin K epoxide reductase complex subunit 1 gene) and *CYP2C9*, have been incorporated into dosing algorithms such as those developed by the International Warfarin Pharmacogenetics Consortium (IWPC) to improve dose prediction and reduce variability in anticoagulant response [168]. However, subsequent clinical trials, including EU-PACT (European Pharmacogenetics of Anticoagulant Therapy) [169] and COAG (Clarification of Optimal Anticoagulation through Genetics) [170], demonstrated that the benefits of genotype-guided dosing may vary across patient populations and clinical settings. These findings highlight both the potential and the limitations of personalised treatment strategies and reinforce the need for rigorous validation of AI-assisted prescribing models across diverse populations.

Targeted therapies also illustrate the complexity of therapeutic effects and toxicity. For example, trastuzumab significantly improves outcomes in HER2-positive breast cancer but is associated with a risk of cardiotoxicity, particularly in patients receiving concurrent or prior anthracycline therapy [171,172]. This demonstrates that even highly selective therapies can produce clinically significant off-target or system-level toxicities that must be carefully monitored. Furthermore, such complexities increase further when involving the administration of “antidote” drugs to reduce, for example, the symptoms of established therapeutic drug toxicity. For example, dexrazoxane, which is widely used for reducing anthracycline cardiotoxicity, can also cause different toxic side effects in some categories of cancer patients [173,174,175].

Immune-based therapies provide another important example of variability in clinical outcomes. Immune checkpoint inhibitors, such as anti-PD-1 (programmed cell death protein 1) or anti-CTLA-4 antibodies (cytotoxic T-lymphocyte-associated protein 4), can produce durable therapeutic responses in some patients but are also associated with immune-related adverse events affecting multiple organs, including the skin, gastrointestinal tract, liver, and endocrine system [176]. These toxicities reflect variability in immune activation and highlight the importance of integrating patient-specific factors and biomarkers into treatment decisions.

The relationship between dose and toxicity is further illustrated by paracetamol (acetaminophen), which is widely used and safe at therapeutic doses but can cause severe hepatotoxicity when the recommended doses are exceeded. In this case, toxicity results from the accumulation of a reactive metabolite (N-acetyl-p-benzoquinone imine) when detoxification pathways are saturated, demonstrating how metabolic capacity and exposure thresholds directly influence clinical outcomes [177,178].

In general, many genotypic and other parameters and factors affect the toxicity and efficacy of drugs. In iron chelation therapy, for example, it is estimated that among the most serious toxicities in iron-loaded patients caused by L1 is agranulocytosis, whereas those caused by DFRA include renal damage or failure, and those caused by DF include anaphylactic reactions and neurotoxic effects [51,179]. The causes of the above toxicities, which occur only in a small proportion of the treated population receiving these three chelating drugs, are not known. However, weekly or fortnightly blood counts and creatinine clearance measurements are mandatory for patients treated with L1 and DFRA, respectively. In such toxicity cases, the change in chelating drug(s) in the affected patients offers alternative therapeutic solutions [51,179].

Together, these examples highlight that therapeutic efficacy and toxicity are closely linked and often may arise from the same underlying pharmacokinetic and pharmacodynamic mechanisms, whereas, in many other cases, the toxicity factors are unknown. Overall, incorporating these outcomes into AI-driven models is essential for predicting both treatment benefit and risk, enabling more precise optimisation of therapy at the individual patient level.

## 8. The Role of Diagnostic Criteria and Biomarkers

The outcomes of efficacy, toxicity, and other drug activity parameters and features are, in most cases, evaluated through diagnostic criteria and biomarkers, including molecular and biochemical markers, haematological indices, organ function tests (e.g., liver and renal parameters), and imaging techniques. These measurable endpoints can confirm therapeutic efficacy and allow the early detection of adverse effects, providing essential real-world data for regular clinical patient monitoring and therapeutic decision-making [35,56,57,58,59,61,64,171,180,181].

In a relevant example, recent advances in genomic diagnostic testing have introduced highly comprehensive screening approaches that can assess a large number of inherited diseases simultaneously in the context of reproductive and prenatal medicine. Expanded carrier screening (ECS), typically performed before or during early pregnancy, uses next-generation sequencing technologies to identify carrier status for hundreds of autosomal recessive and X-linked disorders in a single test, independent of patient ethnicity [182,183,184]. In addition, more advanced approaches such as whole-exome and whole-genome sequencing enable the analysis of thousands of genes at once, allowing the detection of a broad spectrum of monogenic diseases, including rare and de novo variants [185]. These technologies are also increasingly applied in pre-implantation genetic testing and prenatal diagnostics, where they support the identification of clinically actionable genetic biomarkers at the embryo or foetal level [186]. Collectively, these genomic platforms show how large-scale biomarker profiling can be integrated into clinical decision-making, providing a foundation for risk stratification, early diagnosis, and increasingly personalised therapeutic planning [187,188].

Beyond single-gene and genome-wide screening approaches, polygenic risk models further extend the clinical use of genomic biomarkers. For example, recent large-scale studies have developed and validated polygenic risk scores for multiple chronic diseases across diverse populations, demonstrating their potential to predict disease susceptibility and support early risk stratification in clinical practice [189]. In parallel, genome-wide analyses have revealed that common diseases, such as type 2 diabetes, are driven by distinct genetic mechanisms, highlighting the presence of biologically heterogeneous disease subtypes that may require different therapeutic strategies [190]. These findings reinforce the concept that genomic data not only inform disease risk but also define disease biology, directly affecting biomarker selection and treatment decisions.

Large-scale analyses based on whole populations further highlight the importance of genetic diversity in clinical interpretation. For example, studies from the All of Us Research Program [139] have shown that the frequency and distribution of pathogenic variants vary across ancestries, revealing important differences in disease risk and underscoring the need for inclusive genomic datasets in personalised medicine [191]. In addition, genomic studies have demonstrated that disease heterogeneity and progression can be driven by dynamic genomic changes over time, as shown in prostate cancer, where tumour evolution affects disease phenotype and treatment response [192].

Beyond genomic and molecular biomarkers, advanced experimental models are increasingly used to provide functional validation of drug response. Organ-on-chip and tumour-on-chip systems can replicate human tissue architecture, disease progression, and therapeutic responses under physiologically relevant conditions [193]. For example, lung cancer chip models have been shown to reproduce tumour growth dynamics and treatment responses, highlighting the importance of microenvironmental context in drug sensitivity [194]. Similarly, organoid-based tumour-on-chip platforms derived from patients enable the assessment of individual drug responses, offering a functional extension of genomic profiling for personalised treatment selection [195].

The introduction of new diagnostic and theranostic agents and techniques further increases the prospect of personalised medicine in many diseases [196,197,198]. For example, monitoring of iron chelation therapy in thalassaemia major patients using the MRI T2* method, which can identify the level of iron load and also the extent of damage in various organs, as well as determination of the levels of serum ferritin, could all help in the selection of appropriate chelation protocols for each thalassaemia major or other iron-loaded patient [29,61,62,63,64,199].

Overall, the introduction of diagnostic and monitoring systems of organ function and drug activity and toxicity, particularly when incorporating extracellular matrix components, microbiome interactions, and microenvironmental, dietary, and other factors, improves the ability to predict drug efficacy and toxicity, supporting their integration into AI-driven personalised medicine frameworks [200,201,202].

## 9. The Role of Statistical Validation Related to Pharmacological and Clinical Input Features

**Figure 3:**
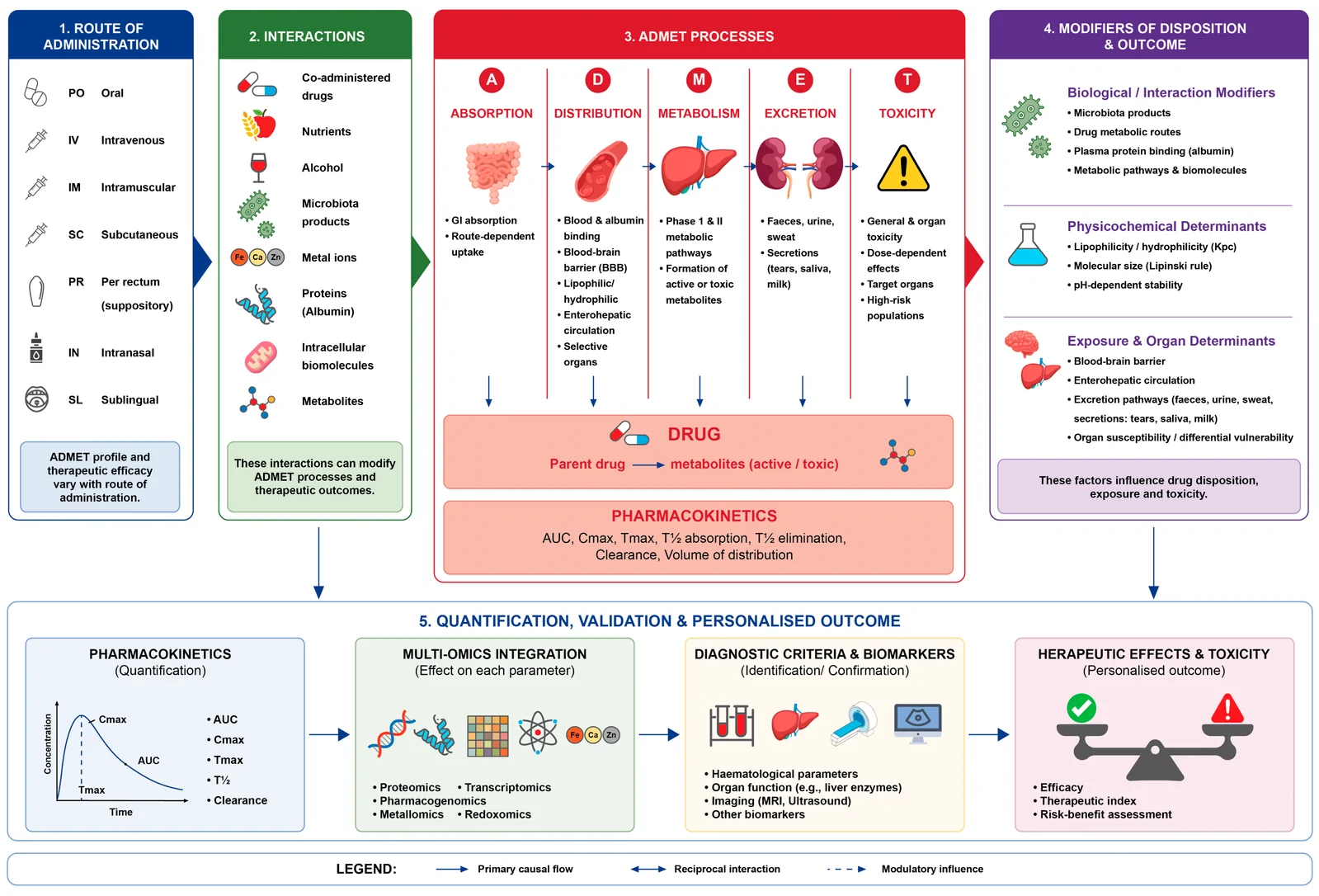
Integrated framework linking route of administration, drug interactions, ADMET processes, and downstream validation factors and parameters to personalised therapeutic outcomes. (Arrows: Solid arrows indicate primary causal flow). (Abbreviations: ADMET: absorption, distribution, metabolism, excretion, toxicity, AUC: area under the curve, BBB: blood–brain barrier, C_max_: maximum concentration, GI: gastrointestinal, IM: intramuscular, IN: intranasal, IV: intravenous, K_pc_: lipid/water partition coefficient, MRI: magnetic resonance imaging, PO: oral, PR: per rectum, SC: subcutaneous, SL: sublingual, T_1/2_: half-life, T_max_: time for maximum concentration).

The pharmacological and clinical input features and parameters described in previous sections could collectively generate high-dimensional datasets. Statistical evaluation and AI modelling should integrate all these inputs to generate predictive and generalisable models applicable to each patient. Key components of these models include assessment of variability, reproducibility, predictive accuracy, sensitivity and specificity, and external validation using independent datasets. Importantly, clinical and experimental data feed back into the system, enabling continuous refinement and updating of the model. Together, these interconnected parameter groups form a coherent, multidimensional framework that supports the development of AI models capable of guiding precise and personalised pharmacotherapy [203,204,205,206]. To further illustrate the integration of these input features into the unified framework, Figure 3 provides a simplified representation of how route of administration, drug interactions, ADMET processes, and downstream validation metrics collectively determine personalised therapeutic outcomes.

In this context, recent advances in AI provide concrete examples of how such complex, heterogeneous datasets can be effectively integrated and translated into clinically useful models. For example, the development of probabilistic histological brain atlases has demonstrated how large-scale, high-resolution datasets can be aligned and analysed to generate biologically meaningful predictions. In a recent study, AI-enabled methods were used to reconstruct three-dimensional histological volumes from thousands of tissue sections and assign probabilistic labels to hundreds of anatomical regions, enabling automated segmentation and disease analysis in MRI datasets [207]. This approach shows how combining multi-scale data with probabilistic modelling and validated ground truth annotations can enhance the accuracy, interpretability, and clinical applicability of AI-driven systems, principles that are directly transferable to personalised pharmacology and drug response prediction.

Computational features for ML applications are summarised in Table 1 and include molecular descriptors, pharmacokinetic factors, multi-omics data, interaction features, clinical variables, imaging-derived metrics, and derived computational representations.

All the above pharmacological and other similar input features for ML models could provisionally be used in personalised medicine, especially in cases of individuals with low drug response or higher than usual toxicity. It is envisaged that ML models using these inputs will routinely be used in an automated manner for all patients in the future and initially in diseases of high priority with high morbidity and mortality rates.

Although numerous reviews have examined the application of AI in drug discovery, pharmacogenomics, clinical decision support, and precision medicine, less attention has been given to how the diverse pharmacological and patient-specific information required for these systems should be systematically organised. The framework presented in this review brings together physicochemical, pharmacokinetic, pharmacodynamic, molecular, clinical, and diagnostic factors and parameters into a single conceptual model that reflects how these factors and parameters interact to influence therapeutic decision-making in individual patients. Rather than viewing these factors and parameters as separate sources of information, it considers them as interconnected components that collectively influence therapeutic decision-making. Although this framework remains conceptual and requires prospective validation, it provides a structured basis for the future development of more transparent, biologically informed, and clinically interpretable AI systems for personalised pharmacotherapy.

## 10. Computational, Statistical and Regulatory Requirements for AI-Assisted Drug Selection

**Figure 4:**
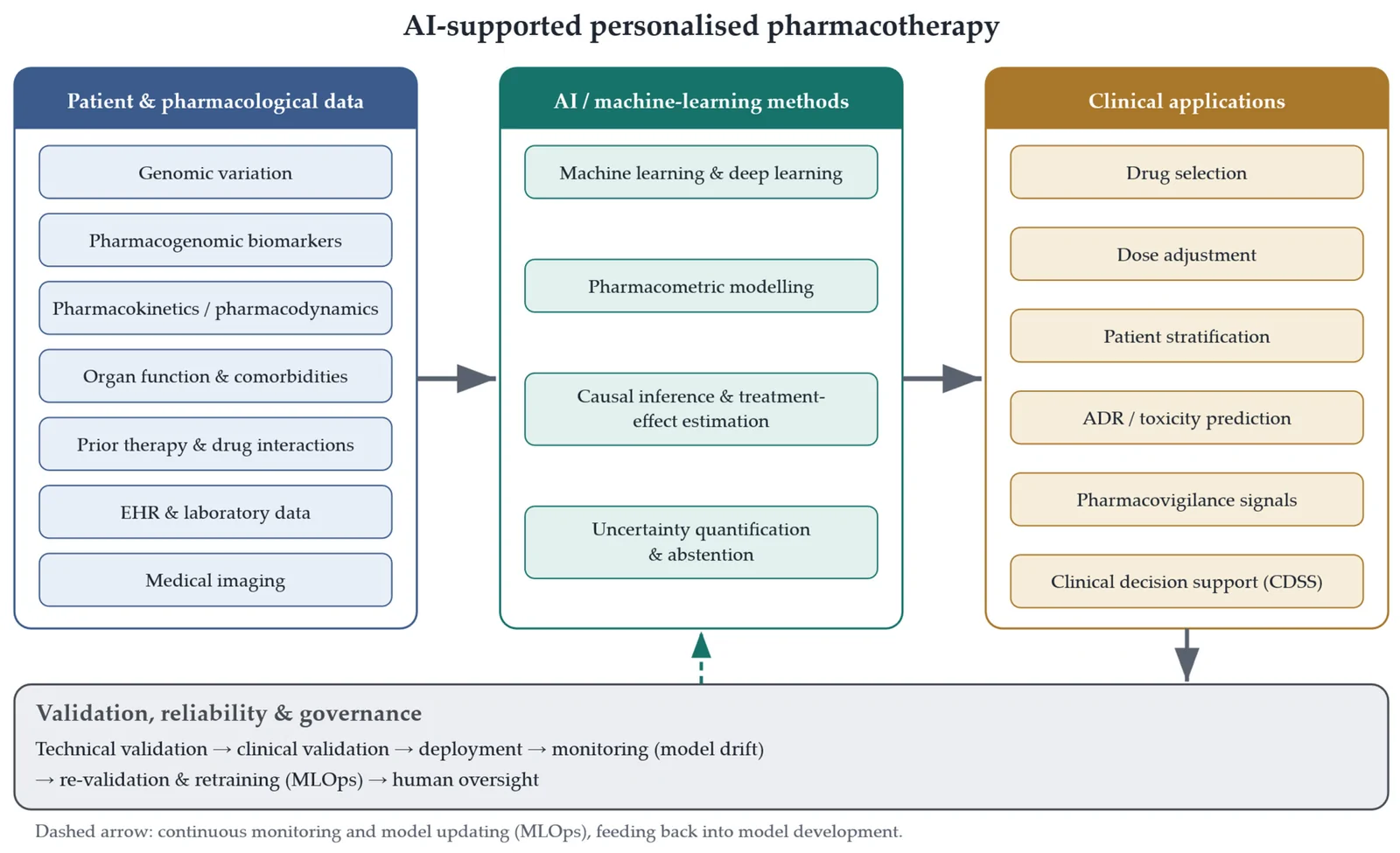
Integrated framework linking patient and pharmacological data, artificial intelligence and machine-learning methods, and clinical applications to personalised therapeutic outcomes, all operating under continuous validation, reliability, and governance, with monitoring and retraining (MLOps) feeding back into model development. (Arrows: Dashed arrow indicates continuous monitoring and model updating (MLOps), feeding back into model development). (Abbreviations: ADR: adverse drug reaction, AI: artificial intelligence, CDSS: clinical decision support system, EHR: electronic health record, MLOps: machine-learning operations).

The preceding sections describe a large number of pharmacological, clinical and molecular quantities that jointly determine drug response, from physicochemical properties and formulation through ADMET processes, drug interactions and molecular targets to diagnostic biomarkers. Using them computationally requires confronting three facts: their number is large relative to the number of patients in whom outcomes are observed, many are missing in routine care, and the comparison of interest is between treatments rather than between patients. Throughout this review, the term “input features and variables” refers to the quantities supplied to a model, as distinct from model parameters, which are the internal weights learned during training. This section sets out the constraints that follow, and Section 11 proposes an architecture in which each is addressed by an explicit design decision. The principal AI and machine learning methods relevant to this discussion are summarised in Figure 4.

Established pharmacometric approaches already address part of this problem. Pharmacokinetic and pharmacodynamic modelling, population pharmacokinetics and Bayesian adaptive dosing describe the exposure and response relationships set out in Section 5 mechanistically, are more data-efficient than ML, and are already validated in therapeutic drug monitoring [79,209]. Where the relationship between dose, exposure and effect is well characterised, these methods remain preferable. Machine learning offers value in a different setting: when candidate determinants are numerous and heterogeneous in type, and no tractable mechanistic model links them to outcome [218]. Selection among candidate drugs differing in target, metabolism and toxicity profile, as in the chelation and antiplatelet examples above, is such a setting.

The first constraint is dimensional. Integrating the multi-omic, clinical and imaging-derived variables described in Section 6, Section 7 and Section 8 at the individual patient level produces datasets in which candidate predictors may outnumber patients with observed outcomes. Under these conditions, models fit noise, and apparent performance in development data does not transfer. Sample size requirements are frequently not met and are larger for machine learning than for regression [219], so regularisation and explicit feature selection are necessary rather than optional. The second constraint concerns data quality. In electronic health records, a value is often missing because a clinician did not consider the test necessary, so missingness is itself informative. Variables recorded after a treatment decision may encode the outcome, producing target leakage, and the serious toxicities described in Section 7 are rare, so class imbalance is the normal condition. The third concerns evaluation, where discrimination alone is insufficient, since a model whose ranking is adequate but whose probabilities are poorly calibrated will mislead at any decision threshold. Calibration and clinical utility should be reported alongside the measures listed in Table 1.

A further constraint is more fundamental. Choosing between treatments requires an estimate of what would happen to a given patient under each option, whereas a model trained on prescribing records estimates what has happened to patients who received each option. These differ because the reason a drug was chosen is usually related to prognosis. Confounding by indication of this kind is reproduced rather than corrected by correlational models, so a system trained naively on such data will tend to recapitulate existing prescribing rather than improve upon it. Methods developed to address this, including target trial emulation, specify the intended comparison explicitly and state the assumptions under which it is identified [220]. Their agreement with randomised evidence is moderate rather than complete [221], which is a reason for caution in interpretation rather than for abandoning the approach.

Even a well-specified model must be shown to work outside its development setting. A system developed on one country’s prescribing patterns, laboratory reference ranges and demographic mix may not transfer to another, making external validation across sites and populations a precondition for deployment. The consequences of omitting it are illustrated by the Epic Sepsis Model [222], deployed at hundreds of hospitals on the basis of internally derived performance estimates, which on independent evaluation showed substantially poorer discrimination than reported, missed most patients who developed sepsis, and generated alerts across a large proportion of admissions. In prescribing support, the equivalent failure would produce unreliable recommendations and alert fatigue. Performance also decays as practice and populations change, so monitoring, periodic revalidation, versioning and retraining under defined governance are required.

Reporting guidance exists for successive stages of this pathway, from model development under TRIPOD+AI (Transparent Reporting of a Multivariable Prediction Model for Individual Prognosis or Diagnosis plus Artificial Intelligence) [217], to early live clinical evaluation including human factors under DECIDE-AI (Developmental and Exploratory Clinical Investigation of DEcision-support systems driven by Artificial Intelligence) [223], to the protocols and reports of subsequent randomised trials under SPIRIT-AI (Standard Protocol Items: Recommendations for Interventional Trials–Artificial Intelligence) and CONSORT-AI (Consolidated Standards of Reporting Trials–Artificial Intelligence) [224,225], the Checklist for Artificial Intelligence in Medical Imaging (CLAIM) applies specifically to imaging studies [226]. A prescribing support tool would need to progress through the same stages, since reported discrimination is not in itself evidence of improved prescribing.

These requirements are increasingly codified in regulation. Governance defines the rules, responsibilities and safeguards under which a system is designed, tested, deployed and updated, covering accountability, data provenance, auditability and human oversight [227]. In the United States, a decision support tool meeting the definition of a medical device is regulated as Software as a Medical Device, and the Predetermined Change Control Plan mechanism allows planned model modifications, together with the methods used to validate them, to be authorised in advance [228], which converts continuous updating into an auditable process. In the European Union (EU), AI systems that are medical devices under Regulation (EU) 2017/745 are classified as high risk under the Artificial Intelligence Act, with obligations covering risk management, data governance, logging, human oversight and post-market monitoring [229,230]. Data used for model development and monitoring remain subject to the General Data Protection Regulation [231]. Representative software platforms, technical resources and pharmacological knowledge bases relevant to implementation are summarised in Table 2.

## 11. A Conceptual Model for AI-Assisted Personalised Drug Selection

**Figure 5:**
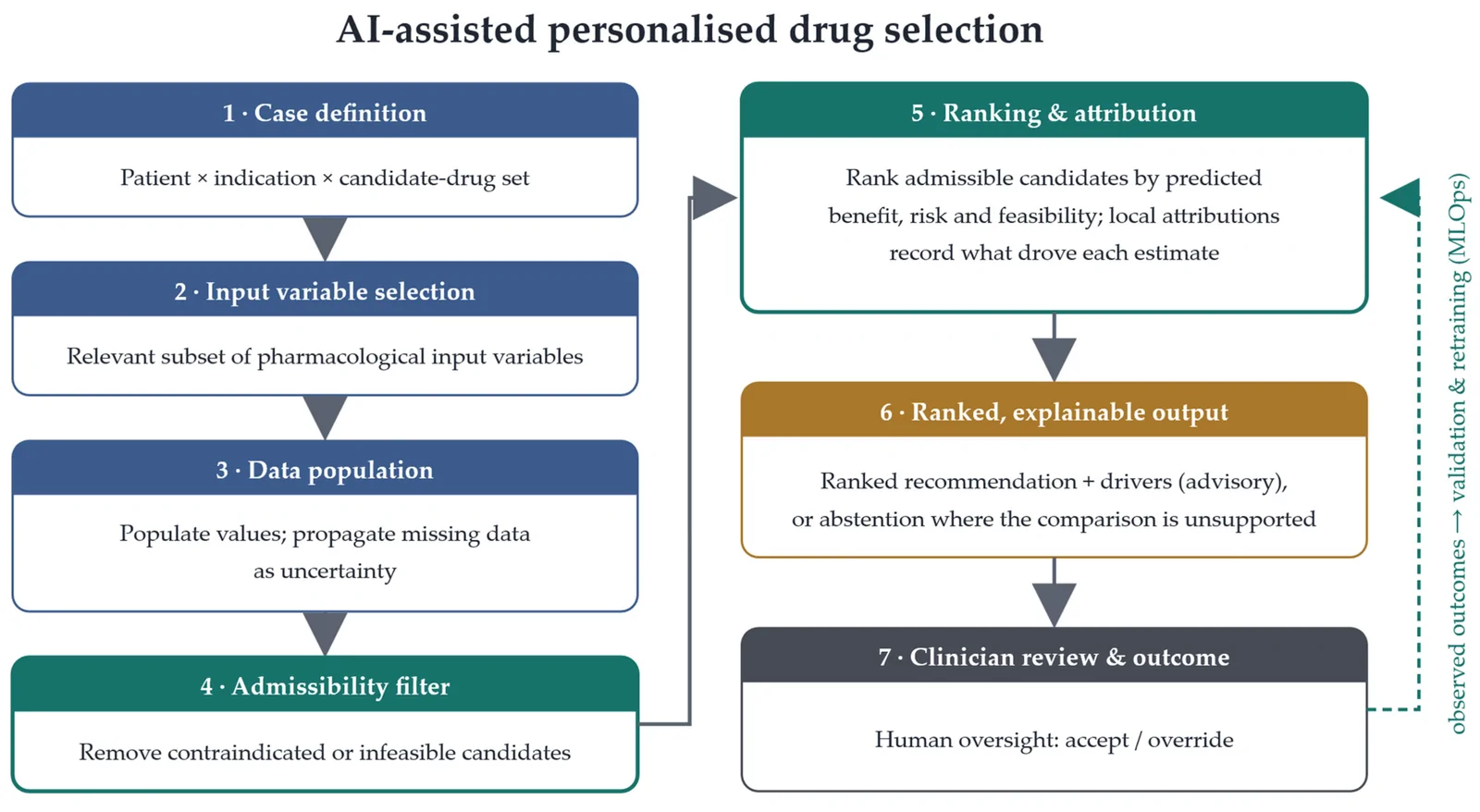
Conceptual model for AI-assisted personalised drug selection. For a defined case (patient, indication, and candidate drugs), the relevant subset of pharmacological input variables is selected and populated with patient- and drug-specific data, with missing values propagated as uncertainty and abstention where the available information does not support a comparison. Clinical guardrails then act as an admissibility filter, removing candidates that are contraindicated or infeasible, and the admissible candidates are ranked by predicted benefit, risk and feasibility, with local attributions recording which input variables drove each estimate. The output is a ranked, explainable recommendation that remains advisory and subject to clinician review, with observed outcomes feeding back into validation and periodic model updating. (Arrows: Dashed arrow indicates that observed outcomes feed back into validation and model updating, as described in Section 10). (Abbreviations: MLOps: machine-learning operations).

The preceding section sets out what an AI-assisted approach to drug selection must contend with. This section proposes an architecture in which each of those constraints is met by an explicit design decision rather than assumed away. The task addressed is selection among a set of candidate drugs specified by the clinician for a defined patient and indication. It is not dose individualisation, for which the pharmacometric methods described above remain better suited. The model presented is a proposed architecture rather than a system that has been implemented, and its purpose is to make the reasoning behind a recommendation inspectable (Figure 5).

For a defined case, comprising the patient, the clinical indication and a set of candidate drugs, the algorithm identifies the input variables relevant to that decision and populates them with patient- and drug-specific values where these are known. Clinical guardrails then remove candidates that are contraindicated or otherwise infeasible, and the remaining candidates are scored and ranked, with the basis of each estimate recorded alongside the recommendation. The output is clinical decision support rather than clinical decision-making and remains subject to clinician review [232].

To illustrate, consider a patient requiring antiplatelet therapy after percutaneous coronary intervention. The relevant input variables include *CYP2C19* genotype, concomitant proton pump inhibitor use, renal function, bleeding risk and prior therapy. Where genotype is available and indicates reduced enzyme activity, the predicted effectiveness of clopidogrel falls relative to alternative agents, and pharmacogenomic status and concomitant medication contribute more strongly to the estimate than other variables [94]. Where a genotype has not been determined, its absence is carried forward as uncertainty rather than imputed silently. The example is given to illustrate the operation of the framework and not as a validated clinical implementation.

The treatment of incomplete data requires specification rather than assertion [233]. Where a variable is absent, single imputation followed by prediction as though the value were known understates uncertainty and can yield confident recommendations from sparse records. Two approaches are appropriate. Multiple imputation generates several completed datasets and pools the resulting predictions so that the variability introduced by imputation is carried into the final estimate. Conformal prediction offers a complementary, model-agnostic route, producing prediction sets with a user-specified coverage probability without assuming a particular data distribution and has been demonstrated for drug response prediction from transcriptomic profiles [234]. The guarantee it provides is marginal rather than conditional, so coverage holds on average across cases rather than for every individual patient.

Both approaches support an explicit abstention policy. Where the missing variables are those on which the comparison between candidates depends, or where no comparable patient in the available data has received one of the candidate drugs, the appropriate output is a statement that the available information does not support a ranking, rather than a ranking accompanied by a wide interval. The second of these conditions is the positivity requirement described in the previous section. The capacity to withhold a recommendation is a safety property of clinical decision support rather than a limitation of it [235].

The prioritisation stage is where case-specific reasoning enters the architecture, and every subsequent step depends on its output. Its premise is that the relative importance of an input variable is not fixed across patients. For example, renal function may dominate one decision and be largely irrelevant to the next. The stage is specified in two parts, which differ in both function and authority.

The first is an admissibility filter. Absolute contraindications, documented intolerance to a route of administration, and the infeasibility of mandatory monitoring act as constraints on the candidate set rather than as quantities to be traded off. A patient with previous L1-induced agranulocytosis receives no L1 recommendation rather than a low-ranked one, and a patient who cannot tolerate injections has parenteral options removed rather than penalised [236,237]. Encoding such rules as constraints, rather than as heavily weighted variables, keeps categorical clinical decisions outside the statistical model, where they cannot be outweighed by a sufficiently favourable prediction.

The second ranks the admissible candidates by their estimated benefit, risk and feasibility, accompanied by local attributions reporting which input variables drove each estimate [238,239]. The estimates on which this ranking depends should themselves be derived under a design capable of supporting a treatment comparison, such as a target trial emulation [220], rather than from prescribing records analysed as though treatment assignment were unrelated to prognosis. These attributions are diagnostic rather than generative: they describe the estimate rather than produce it, and their function is auditability rather than accuracy. Their limitations should be stated. Attribution values are unstable across resampling and across choices of background distribution; they allocate credit unreliably among correlated predictors, a pervasive condition in pharmacological data where genotype, metabolic phenotype and exposure are strongly co-dependent, and they carry no causal interpretation. An attribution indicates what the model responded to, not what would follow from altering the variable.

The relationship between the two parts is itself informative. Where the constraint layer removes a candidate that the ranking layer would have placed first, the system has identified a case in which statistical evidence and clinical rule diverge. Presenting that divergence to the clinician, rather than resolving it silently, is the property that distinguishes this architecture from a predictive model with an explanation attached.

The admissible candidates are then combined into a drug-specific predicted benefit and feasibility profile, based on structured benefit-risk methodologies implemented at the individual patient level [240,241]. They are presented to the clinician as a ranked recommendation, including their sensitivity to the input variables and a confidence measure, allowing for review and override where needed [242,243]. Observed outcomes feed back into validation and periodic model updating, addressing the performance decay described in the previous section.

The framework should be regarded as a conceptual architecture rather than a validated system. The model parameters, including those of the predictive model from which the input-variable weights are derived, would require a clinical study in an appropriate patient population for their development and validation, as would the ethical and regulatory approvals that such work entails [244]. Its principal limitations follow from this. The quality of a recommendation would depend on the representativeness of the training data and on the appropriateness of the underlying estimates, and it is envisaged as a supportive rather than an exclusive decision aid. A proof-of-concept implementation in a single, well-characterised indication would be the appropriate next step. Such a study would test feasibility rather than establish clinical utility, which would require prospective evaluation against current prescribing practice, with comparator and outcome measures specified in advance.

## 12. Conclusions

Artificial intelligence is rapidly changing the landscape of pharmacology, drug development, and clinical therapeutics, creating new opportunities for more precise and individualised treatment. However, the success of AI in personalised medicine will ultimately depend not on the sophistication of the algorithms themselves, but on the quality, completeness, and reliability of the pharmacological and clinical knowledge that they incorporate. Throughout this review, from a pharmaceutics perspective, it has been highlighted that drug response is determined by a complex network of interacting factors, including physicochemical properties, formulation, routes of administration, drug interactions, pharmacokinetics, pharmacodynamics, ADMET characteristics, molecular targets, multi-omics data, biomarkers, patient-specific clinical characteristics, and therapeutic outcomes. None of these factors acts in isolation, and their relative importance varies considerably between individual patients. Thus, AI-assisted personalised medicine depends on the integration of these factors into clinically meaningful decision-support frameworks.

Realising this potential will also require pharmacological knowledge resources to develop incrementally. Initial efforts are likely to focus on well-characterised drugs, therapeutic areas, and existing pharmacogenomic and clinical datasets before progressively expanding to more complex multimodal data sources. Interpretation of AI-assisted treatment recommendations also requires recognition that treatment selection differs from conventional prediction because the outcomes of alternative treatments for an individual patient are not directly observable.

Despite the progress of AI, it is unlikely at this stage to be able to replace human judgement in pharmacotherapy and medicine. Artificial intelligence generates recommendations from the information on which it has been trained, and these are not inherently peer-reviewed or independently verified and therefore should not be accepted uncritically. Clinical decision-making requires scientific reasoning, professional experience, ethical judgement and consideration of patient-specific circumstances that extend beyond the capabilities of current systems. For this reason, AI should remain a decision-support tool for now, with final responsibility for therapeutic decisions resting with physicians, pharmacists and other healthcare professionals.

The future of AI in pharmacotherapy will therefore depend not only on advances in computational methods but also on the continued expansion of pharmacological knowledge, comprehensive mapping of drug- and metabolite-related parameters, rigorous validation, transparent governance, and close collaboration between pharmacologists, clinicians, computational scientists, and regulatory authorities. The continued development and integration of pharmacological knowledge resources will provide an important foundation for trustworthy AI systems capable of supporting safer drug development, more accurate therapeutic decision-making, and the broader implementation of precision personalised medicine.

## Figures and Tables

**Table 1:** Pharmacological and computational factors and parameters relevant to AI-assisted personalised drug selection.

Category	Factor Group	Specific Features
Drug-specific and molecular properties [36,208]	Structural and chemical descriptors	SMILES representations, 2D/3D molecular graphs, molecular structure features
	Physicochemical descriptors	Molecular fingerprints, RDKit descriptors, lipophilicity-related features
	Drug activity predictors	Binding affinity, QSAR features, drug-likeness parameters
	ADMET-related properties	Absorption, distribution, metabolism, excretion, toxicity predictors
	Nanomedicine properties	Particle size, zeta potential, polydispersity index
Pharmacokinetics and exposure [79,112,209]	Drug disposition parameters	Concentration–time profiles, clearance, bioavailability
	Dose–exposure relationships	Dose–response curves, therapeutic concentration ranges
	Distribution-related features	Tissue distribution, permeability, plasma protein binding
	Formulation-related kinetics	Drug release kinetics, delivery system behaviour
Multi-omics data [135,210]	Genomics	SNPs, genetic variants, resistome profiles
	Transcriptomics	Gene expression data, RNA-sequencing outputs
	Proteomics	Protein abundance, signalling pathways
	Metabolomics	Pathway analysis
	Systems biology features	Gene regulatory networks, pathway activity data
	Integrated omics	Multi-omics datasets and cross-layer interactions
Drug–biological system interactions [126,211]	Molecular interactions	Protein–ligand binding, docking predictions
	Functional biological effects	Enzyme modulation, receptor binding
	Cellular responses	Responder vs. non-responder classification
	Microbial interactions	Antimicrobial resistance mechanisms, resistance predictors
Patient-specific clinical factors and parameters [212,213]	Clinical biomarkers	Diagnostic biomarkers, disease markers
	Physiological features	Organ function indicators (mainly liver, kidney)
	Patient stratification features	Disease severity, phenotype classification, responder vs. non-responder classification
	Clinical datasets	Epidemiological and real-world patient data
Imaging and phenotypic data [214,215]	Imaging-derived features	Medical imaging features, tissue segmentation data
	Advanced spatial data	Spatial transcriptomics, tissue mapping
	Experimental models	Organoid responses, phenotypic screening outcomes
Feature engineering and data transformation [216]	Feature extraction	Omics-derived features, molecular descriptors
	Dimensionality reduction	PCA, *t*-SNE, UMAP latent variables
	Feature selection outputs	LASSO-selected features, random forest importance
	Data representations	Embeddings and latent feature spaces
Outcome and validation factors [203,217]	Therapeutic outcomes	Drug efficacy, treatment response
	Safety endpoints	Toxicity profiles, adverse effects
	Resistance outcomes	Drug resistance or AMR prediction outputs
	Model evaluation metrics	AUROC, precision, recall, F1-score, Brier score
	Validation datasets	Internal and external validation data

Table 1 includes representative examples of input variables, analytical approaches, and model evaluation metrics relevant to AI-assisted personalised drug selection. These categories are presented together for conceptual completeness and do not imply that all listed elements are pharmacological parameters and factors. Abbreviations: ADMET: Absorption, Distribution, Metabolism, Excretion and Toxicity, AMR: Antimicrobial Resistance, AUROC: Area Under the Receiver Operating Characteristic Curve, LASSO: Least Absolute Shrinkage and Selection Operator, PCA: Principal Component Analysis, QSAR: Quantitative Structure–Activity Relationship, RDKit: Molecular Descriptor Toolkit, SMILES: Simplified Molecular Input Line Entry System, SNPs: Single Nucleotide Polymorphisms, *t*-SNE: *t*-distributed Stochastic Neighbor Embedding, UMAP: Uniform Manifold Approximation and Projection.

**Table 2:** Selected technical and pharmacological resources relevant to the implementation of AI in pharmacology and medicine.

Area	Example	Description	Use in Pharmacology	Link
Pharmaco-genomic knowledge	PharmGKB	Curated knowledge base of variant–drug associations with graded levels of evidence	Identifying and grading pharmacogenomic input variables	https://www.pharmgkb.org
Genotype-based prescribing guidance	CPIC	Peer-reviewed, periodically updated genotype-directed prescribing guidelines	Encoding genotype-directed prescribing rules and clinical guardrails	https://cpicpgx.org/
Drug, target and interaction data	DrugBank	Curated knowledge base of drugs, targets, pharmacology and interactions	Drug properties, targets and interaction data for candidate assessment	https://go.drugbank.com/
Bioactivity data	ChEMBL	Manually curated open database of bioactive molecules	Structure–activity and target-affinity variables	https://www.ebi.ac.uk/chembl/
Adverse effect data	SIDER	Side effect resource compiled from package inserts and public documents; content not updated since 2015	Baseline adverse-effect associations, subject to currency limitations	https://sideeffects.embl.de/
Pharmaco-vigilance data	AEMS (formerly FAERS)	Spontaneous adverse event and medication error reports supporting post-marketing surveillance	Safety-signal detection and toxicity endpoints	https://open.fda.gov/data/faers
Model explainability	SHAP	SHapley Additive exPlanations	Showing how variables contribute to model outputs	https://shap.readthedocs.io/
Medical imaging reporting	CLAIM	Checklist for Artificial Intelligence in Medical Imaging	Reporting medical image-analysis studies	https://doi.org/10.1148/ryai.240300
Prediction-model reporting	TRIPOD+AI	Transparent Reporting of a Multivariable Prediction Model for Individual Prognosis or Diagnosis plus Artificial Intelligence	Reporting prediction models that use regression or machine learning	https://doi.org/10.1136/bmj-2023-078378

Resources were included if they are openly accessible or publicly documented, are actively maintained or have a documented final release, have a citable methodological description, and support one of the functions discussed in this section. The list is illustrative rather than exhaustive. All URLs were finally accessed on 30 August 2026. Abbreviations: AEMS: FDA Adverse Event Monitoring System (formerly FDA Adverse Event Reporting System, FAERS), AI: Artificial Intelligence, CLAIM: Checklist for Artificial Intelligence in Medical Imaging, CPIC: Clinical Pharmacogenetics Implementation Consortium, EHR: Electronic Health Record, SHAP: SHapley Additive exPlanations, TRIPOD+AI: Transparent Reporting of a Multivariable Prediction Model for Individual Prognosis or Diagnosis plus Artificial Intelligence.

## Data Availability

No new data were created or analysed in this study.

## Abbreviations

The following abbreviations are used in this manuscript:

ADMET	Absorption, Distribution, Metabolism, Excretion and Toxicity
ADR	Adverse Drug Reaction
AI	Artificial Intelligence
AMR	Antimicrobial Resistance
AUC	Area Under the Curve
AUROC	Area Under the Receiver Operating Characteristic Curve
BBB	Blood–Brain Barrier
CDSS	Clinical Decision Support System
CLAIM	Checklist for Artificial Intelligence in Medical Imaging
CONSORT-AI	Consolidated Standards of Reporting Trials–Artificial Intelligence
CTLA-4	Cytotoxic T-Lymphocyte-Associated Protein 4
CYP	Cytochrome P450
DECIDE-AI	Developmental and Exploratory Clinical Investigation of DEcision-support systems driven by Artificial Intelligence
DF	Deferoxamine
DFRA	Deferasirox
ECS	Expanded Carrier Screening
EHR	Electronic Health Record
ER	Oestrogen Receptor
GI	Gastrointestinal
HER2	Human Epidermal Growth Factor Receptor 2
IM	Intramuscular
IN	Intranasal
INR	International Normalised Ratio
IV	Intravenous
IWPC	International Warfarin Pharmacogenetics Consortium
Kpc	Lipid/Water Partition Coefficient
L1	Deferiprone
LASSO	Least Absolute Shrinkage and Selection Operator
LD_50_	Lethal Dose 50
LLM	Large Language Model
logP	Logarithm of the Partition Coefficient
ML	Machine Learning
MLOps	Machine Learning Operations
MRI	Magnetic Resonance Imaging
PCA	Principal Component Analysis
PD-1	Programmed Cell Death Protein 1
P-gp	P-glycoprotein
PO	Oral
PR	Per Rectum
QSAR	Quantitative Structure–Activity Relationship
RDKit	Molecular Descriptor Toolkit
SC	Subcutaneous
SHAP	SHapley Additive exPlanations
SL	Sublingual
SMILES	Simplified Molecular Input Line Entry System
SNP	Single Nucleotide Polymorphism
SPIRIT-AI	Standard Protocol Items: Recommendations for Interventional Trials–Artificial Intelligence
t_1/2_	Half-life
Tmax	Time for Maximum Concentration
TNBC	Triple-Negative Breast Cancer
TRIPOD+AI	Transparent Reporting of a Multivariable Prediction Model for Individual Prognosis or Diagnosis plus Artificial Intelligence
*t*-SNE	*t*-distributed Stochastic Neighbor Embedding
UMAP	Uniform Manifold Approximation and Projection
VKORC1	Vitamin K epoxide reductase complex subunit 1 gene
